# Effect of Cyclic Mechanical Stimulation on the Expression of Osteogenesis Genes in Human Intraoral Mesenchymal Stromal and Progenitor Cells

**DOI:** 10.1155/2014/189516

**Published:** 2014-04-07

**Authors:** Birgit Lohberger, Heike Kaltenegger, Nicole Stuendl, Michael Payer, Beate Rinner, Andreas Leithner

**Affiliations:** ^1^Department of Orthopaedic Surgery, Medical University of Graz, Auenbruggerplatz 5, 8036 Graz, Austria; ^2^Department of Oral Surgery and Radiology, School of Dental Medicine, Medical University of Graz, Auenbruggerplatz 5, 8036 Graz, Austria; ^3^Center for Medical Research, Medical University of Graz, Auenbruggerplatz 5, 8036 Graz, Austria

## Abstract

We evaluated the effects of mechanical stimulation on the osteogenic differentiation of human intraoral mesenchymal stem and progenitor cells (MSPCs) using the Flexcell FX5K Tension System that mediated cyclic tensile stretch on the cells. MSPCs were isolated from human mandibular retromolar bones and characterized using flow cytometry. The positive expression of CD73, CD90, and CD105 and negativity for CD14, CD19, CD34, CD45, and HLA-DR confirmed the MSPC phenotype. Mean MSPC doubling time was 30.4 ± 2.1 hrs. The percentage of lactate dehydrogenase (LDH) release showed no significant difference between the mechanically stimulated groups and the unstimulated controls. Reverse transcription quantitative real-time PCR revealed that 10% continuous cyclic strain (0.5 Hz) for 7 and 14 days induced a significant increase in the mRNA expression of the osteogenesis-specific markers type-I collagen (Col1A1), osteonectin (SPARC), bone morphogenetic protein 2 (BMP2), osteopontin (SPP1), and osteocalcin (BGLAP) in osteogenic differentiated MSPCs. Furthermore, mechanically stimulated groups produced significantly higher amounts of calcium deposited into the cultures and alkaline phosphatase (ALP). These results will contribute to a better understanding of strain-induced bone remodelling and will form the basis for the correct choice of applied force in oral and maxillofacial surgery.

## 1. Introduction

Mesenchymal stem and progenitor cells (MSPCs) are promising candidates for cellular therapy in bone repair and regeneration of degenerative diseases due to their accessibility, expandability, and multipotent differentiation potential [1–6]. Bone marrow (BM) is regarded as the main source of MSPCs for experimental and clinical application [6], but due to the limited number of BM-MSPCs available for autogenous use, the implementation of alternative sources of MSPCs is particularly important. In a previous work, we identified intraoral tissues as potential sources of multipotent progenitor cells for tissue engineering approaches [7]. A major goal in implantology is the development of minimally invasive techniques that allow predictable alveolar crest reconstruction as well as reconstruction of critical size defects resulting from resorption, trauma, cancer, or metabolic disorders. Various techniques using mainly autogenous bone grafts alone or in combination with bone substitutes have, with varying degrees of success and with limitations mainly in regard to donor site morbidity, been introduced and established in daily practice [8–10].

It has been well documented that the bone remodelling process is initiated by the sensing of mechanical stimuli by osteocytes which in turn signal osteoblasts to form the bone matrix. By applying what is known about physiological conditions, it is reasonable to deduce that the same mechanical stimulation may play a role in the differentiation of MSPCs down an osteogenic pathway. Utilizing the principle of mechanically stimulating bone formation, uniaxial tensile strain was successfully used to induce bone regeneration via distraction osteogenesis [11–14]. Undifferentiated human MSPCs are highly sensitive to cyclic tensile strain which transcriptionally controls early osteochondrogenic response* in vitro* [15]. Strain alone can induce a significant increase in bone morphogenetic protein 2 (BMP2) mRNA levels in human BM-MSPCs without any addition of osteogenic supplements [16]. However,* in vivo* bone healing is much more effective when osteogenically differentiated cells are transplanted into the bone defect rather than undifferentiated MSPCs [17].

In this study, we used the FX5K Tension System to assess the effects of mechanical strain on* in vitro* differentiation to osteoblast-like cells, as well as the expression of osteogenesis-related transcription factors. Undifferentiated and osteogenic differentiated human intraoral MSPCs were seeded into BioFlex plates and cultivated statically or dynamically over a 14-day period. Differentiation was assessed using reverse transcription quantitative real-time PCR for the runt-related transcription factor 2 (RUNX2), the early osteogenic markers alkaline phosphatase (ALPL), bone morphogenetic protein 2 (BMP2), type-I collagen (Col1A1), and osteonectin (SPARC), and the osteogenic late stage markers osteocalcin (BGLAP) and osteopontin (SPP1).

The investigation of the effect of mechanical stimulation on human intraoral MSPCs provided insight into the mechanisms of bone regeneration, which play a major role in oral and maxillofacial surgery.

## 2. Material and Methods

### 2.1. Intraoral Tissue Harvest and Cell Culture

Explant MSPC cultures were established from intraoral tissue samples of posterior maxilla as well as from mandibular retromolar bone harvested during routine oral surgical interventions (wisdom tooth removal, augmentation procedures, and implantation). The study protocol was approved by the local ethics committee, and informed consent was obtained from each oral surgery patient. A total of ten patients, seven female and three male, aged between 15 and 47 were included in the study. Patients with metabolic bone diseases, local inflammatory processes, and impaired blood coagulation and pregnant women were excluded. The harvesting procedure was performed under sterile conditions with local anaesthesia (Ultracain Dental Forte, Maxilla; Sanofi-Aventis, Vienna, Austria) using a trephine burr 3.8 mm in diameter and 11 mm in length. The harvested bone samples were between 4 and 6 mm in length and showed cortical or cortical and cancellous structure. The obtained bone samples were rinsed extensively with phosphate-buffered saline (PBS; PAA Laboratory, Pasching, Austria) and cleaned with sharp instruments under the 10-fold magnification of a light microscope. After the cleaning procedure, the biopsies were transferred into 75 cm^2^ culture flasks (TPP, Trasadingen, Switzerland) with an appropriate volume of culture medium and incubated in a humidified atmosphere with 5% CO_2_ at 37°C for cell isolation and expansion.

### 2.2. Cell Culture and Long-Term Expansion

MSPCs were cultured in *α*-modified minimum essential medium (*α*-MEM; Sigma-Aldrich, Vienna, Austria) and supplemented with 10% pooled human platelet lysate (pHPL) [18] after the addition of 2 U/mL stabilisator-free heparin (Biochrom AG, Berlin, Germany), 2% penicillin-streptomycin (GIBCO Invitrogen, Darmstadt, Germany), 0.5% L-glutamine (GIBCO Invitrogen), 0.2% amphotericin B (PAA Laboratory), and 2.5% HEPES buffer (Sigma-Aldrich, Vienna, Austria). Total MSPC cell number and doubling time were evaluated during cell expansion and MSPSs were then cultured with a reduced seeding density technique for three additional passages [19].

### 2.3. Flow Cytometry

A total of 1 × 10^5^ MSPCs were resuspended in a final volume of 200 *μ*L PBS for flow cytometric analysis. The commercial monoclonal antibodies CD73 PE, CD90 APC, CD105 PE, CD45 APC-Cy7, CD34 APC, CD14 FITC, CD19 APC, and HLA-DR APC (BD Bioscience, San Jose, CA) were applied for characterization. The optimal amount of each antibody had previously been determined by titration and antibodies with nonoverlapping spectra were combined in two-colour staining panels. Background staining for antibodies was performed in negative cell lines and with matched fluorochrome-conjugated isotype controls. Flow cytometry analysis was performed on a FACS LSR II System (BD Bioscience), and data were acquired using FACSDiva software (BD Bioscience) and analysed with FCS Express software (De Novo Software, Los Angeles, CA). The day-to-day consistency of measurements was checked by Rainbow Beads (BD Bioscience). Viable cells were gated on forward scatter (FSC) and side scatter (SSC) in order to exclude debris and cell aggregates. MSPCs were defined by their phenotype and analysed on a logarithmic scale. Data from all donors were collected under identical parameters and analysed by collecting 10,000 events.

### 2.4. Mechanical Stimulation

The Flexcell FX-5000 Tension System (FX5K; Flexcell International Corp, Hillsborough, NC) was used to apply mechanical cyclic tensile stretch to the MSPCs. The Flexcell FX-5000 is a computer-based system that uses a vacuum to strain cells adhered to flexible silicon membranes (BioFlex plates; Flexcell International Corp) arranged in a format of six wells per plate with a total growth area of 9.62 cm^2^/well and a membrane thickness of 0.05 mm. The deformation of the flexible membrane of the plates also causes the attached cells to deform. Programming the magnitude, duration, and frequency of the negative pressure in the Flexcell apparatus creates desired strain profiles. MSPCs were seeded onto the collagen type-I-coated BioFlex plates at a density of 5 × 10^4^ cells/well. When cultures reached approximately 70% to 80% confluence, undifferentiated (EX) and osteogenic differentiated (OG) MSPCs were subjected to continuous mechanical stimulation with a uniaxial sinusoidal waveform with 10% elongation and a frequency of 0.5 Hz for 7 and 14 days. Each cycle consisted of 10 s strain and 30 s relaxation (Figure 2(a)). Control cultures were grown under the same conditions but without the strain protocol. Control cultures were grown under the same conditions but without the strain protocol.

### 2.5. Osteogenic Differentiation

Intraoral MSPCs used for osteogenic differentiation were derived from the third to sixth passage. MSPCs were seeded at a density of 10^4^ cells/cm^2^ in expansion medium containing Dulbecco's Modified Eagle's Medium (DMEM-F12; GIBCO Invitrogen), 10% FBS (Lonza, Braine-l'Alleud, Belgium), 1% penicillin-streptomycin, 1% L-glutamine, and 0.1% amphotericin B. Osteogenic differentiation was induced over two weeks by shifting the cells to an osteoinductive medium (OG) composed of the expansion medium supplemented with 100 nM dexamethasone, 0.1 mM ascorbic-acid-2-phosphate, and 10 mM *β*-glycerophosphate (all from Sigma-Aldrich). Alkaline phosphatase (ALP) enzyme activity was photometrically determined at day 14 in triplicate with a p-nitrophenyl phosphate liquid substrate system (Sigma-Aldrich) at 405 nm on a microplate reader (BioRad, Vienna, Austria) [20]. For the visualization and quantification of calcium phosphate deposits, Alizarin Red S (ARS) staining was used at days 7 and 14. Cells were fixed with 10% formaldehyde (Merck, Spittal/Drau, Austria) and incubated with a 1% ARS staining solution. Quantitation of ARS staining was performed by elution of the fixed cells with 10% cetylpyridinium chloride (Sigma-Aldrich) measuring absorbance at 570 nm on a microplate reader [21].

### 2.6. Lactate Dehydrogenase Assay

Lactate dehydrogenase (LDH) activity was measured using the CytoTox-ONE Homogeneous Membrane Integrity Assay (Promega, Mannheim, Germany). The amount of fluorescence produced is proportional to the number of lysed cells. After 7 and 14 days of mechanical stimulation, cell culture supernatants were collected and analysed to examine the state of cellular damage. In short, 50 µL of supernatant and 50 µL of working solution were mixed in white 96-well microtiter plates and incubated in the dark at room temperature for 30 minutes. The reaction was terminated by the addition of 50 µL stop solution and fluorescence was measured at 560/590 nm (Fluostar; BMC Labtech, Ortenberg, Germany).

### 2.7. Reverse Transcription Quantitative Real-Time PCR (RT-qPCR)

RT-qPCR was performed in order to determine the relative expression of the early osteogenic markers ALPL, BMP2, Col1A1, RUNX2, and SPARC and the osteogenic late stage markers BGLAP and SPP1. Total RNA was isolated from osteogenic differentiated and undifferentiated control cells with RNeasy Mini Kit (Qiagen, Hilden, Germany) according to the manufacturer's recommended protocol. RNA quality was analysed using the Agilent RNA 6000 Nano Kit and the Bioanalyzer 2100 (Agilent Technologies, Santa Clara, CA). All RIN values were between 9.2 and 10.0. DNA was digested with 1 U DNase (Fermentas, St. Leon-Rot, Germany) per µg RNA. One µg RNA was reverse transcribed using RevertAid cDNA Synthesis Kit (Fermentas). RT-qPCR reactions were performed in triplicate using the Platinum SYBR Green Super Mix with ROX (Invitrogen) on AB7900HT (Applied Biosystems, Invitrogen). The reference genes glyceraldehyde 3-phosphate dehydrogenase (GAPDH), *β*-actin (ACTB), and hypoxanthine phosphoribosyltransferase (hprt-n) were used for normalization and in order to show their stable expression in different tissues [22]. The following primers were used for RT-qPCR: QuantiTect primer assays (Qiagen) for ALPL (ID QT00012957), BMP2 (ID QT00012544), Col1A1 (ID QT00037793), RUNX2 (ID QT00020517), SPARC (ID QT00018620), BGLAP (ID QT00232771), and SPP1 (ID QT01008798). The expression level (*C*_*T*_) of the target gene was normalized to the reference genes (GAPDH, ACTB, and hprt-n) (Δ*C*_*t*_) and then the Δ*C*_*t*_ of the test sample was normalized to the Δ*C*_*t*_ of the controls (ΔΔ*C*_*t*_). Finally, the expression ratio was calculated with the 2^−ΔΔ*C*_*t*_^ method (*P < 0.05) [23].

### 2.8. Statistical Analysis

All values are expressed as mean ± standard deviation (SD). The exact Wilcoxon test was used to evaluate the differences between groups. The exact Friedman test (more than two time points) and the exact paired Wilcoxon test (2 time points) were applied to test for changes between time points. Two-sided *P* values below 0.05 were considered statistically significant. *P* values were not adjusted for multiple comparisons. Graphic data were prepared with SigmaPlot (Systat Software Inc., San Jose, CA).

## 3. Results

### 3.1. Expansion of Human Intraoral MSCPs

Bone explants were successfully harvested during routine oral surgery interventions in all patients (*n* = 10). Evaluation of postoperative pain and patient satisfaction revealed no difference compared to the control group. Cells exhibiting morphologic characteristics of human BM-stromal cells (mononuclear, fibroblast-like, spindle-shaped, and plastic-adherent) were isolated from all samples within 4 to 8 days, independent of donor gender, age, or macroscopic bone explant structure. Confluence was reached after 13 to 15 days. To expand maxillary cells and analyse the cell doubling time, cells were trypsinised and cultivated with a reduced seeding density technique for three additional passages. The doubling time of each culture of passage three was calculated with a two time-point calculation tool. The doubling time (*Td*) of each culture of passage three was calculated with a two time-point calculation formulas *Td* = (*t*_2_ − *t*_1_)∗Log(2)/log⁡(*q*_2_/*q*_1_)(*t*_1_, *t*_2_ = time  points; *q*_1_, *q*_2_ = growing  quantities. The mean doubling time of the MSPCs was 30.4 ± 2.1 hrs. The mean total number of cells after three passages was 1.2 ± 0.2 × 10^7^.

### 3.2. Characterization of Human Intraoral MSCPs by Flow Cytometry

**Figure 1:**
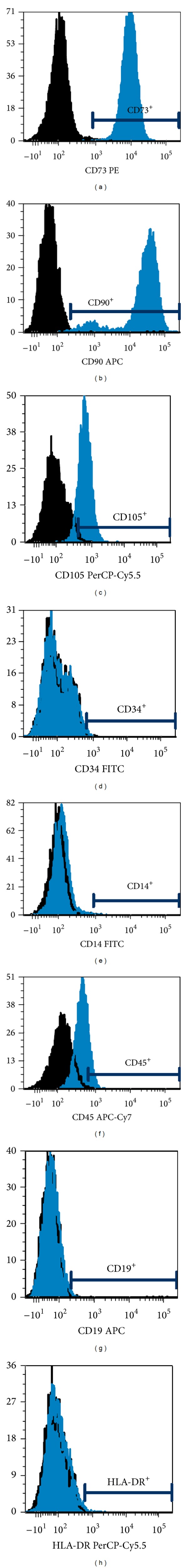
Multicolour flow cytometric immunophenotypic analysis with monoclonal antibodies. Intraoral MSPCs at passages 3–7 were labelled with specific fluorochrome-conjugated monoclonal antibodies against the indicated surface antigens and analysed by flow cytometry. Analysed MSPCs demonstrated the same immunophenotype, with expression of (a) CD73, (b) CD90, and (c) CD105 but no expression of (d) CD14, (e) CD19, (f) CD34, (g) CD45, and (h) HLA-DR. A representative example of ten experiments is shown. PE: phycoerythrin; APC: allophycocyanin; PerCP: peridium-chlorophyll protein complex; FITC: fluorescein isothiocyanate.

Viable cells were gated on the forward/side scatter, and aggregates were excluded with the FSC/W and FSC/A. The positive expression of CD73, CD90, and CD105, low level of CD45, and negativity for CD14, CD19, CD34, and HLA-DR confirmed the phenotype of intraoral MSPCs (Figure 1). Intraoral MSPCs from all patients demonstrated the same immunophenotype. Flow cytometry data are shown in Figure 1 and Table 1.

### 3.3. Intraoral MSPC Morphology and Cellular Activity during Mechanical Stimulation

**Figure 2:**
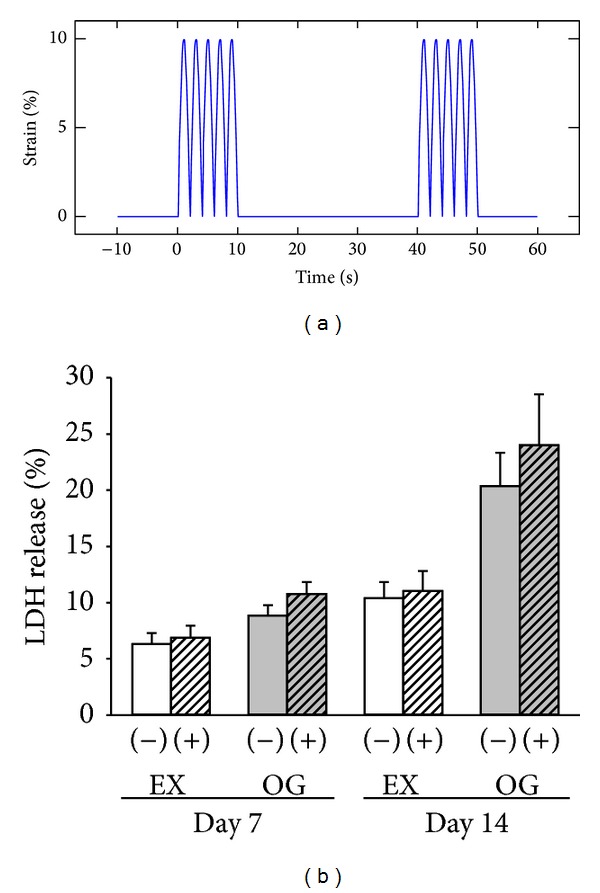
Straining profile and effects of mechanical stimulation on the release of lactate dehydrogenase (LDH) from human intraoral MSPCs. (a) Profile of a uniaxial sinusoidal waveform with 10% elongation and a frequency of 0.5 Hz, whereby each cycle consists of 10 s strain and 30 s relaxation. (b) The percentage of LDH released into the culture media was measured after 7 and 14 days of culture. Each bar represents the mean ± SD of independent experiments performed in triplicate (*n* = 8); *P < 0.05.

The strain profile used in the present study was based on the timing and theorized intensity profiles of common repetitive motion strains that are often the result of masticatory movements (Figure 2(a)). No morphological differences were observed between the mechanically stimulated groups and unstimulated groups. However, the uniform alignment of cells was observed for the mechanically stimulated groups. The percentage of LDH release showed no significant differences between the mechanically stimulated groups (EX(+) and OG(+)) and unstimulated groups (EX(−) and OG(−)) (Figure 2(b)).

### 3.4. Expression of Osteogenesis-Specific Markers Affected by Mechanical Stimulation

To determine the influence of 10% cyclin strain on osteogenesis, undifferentiated (EX) and osteogenic differentiated (OG) human intraoral MSPCs were mechanically stimulated with the Flexcell FX5K Tension System for 14 days. Relative mRNA expression levels of the runt-related transcription factor 2 (RUNX2), the early osteogenic markers type-I collagen (Col1A1), osteonectin (SPARC), bone morphogenetic protein 2 (BMP2), and alkaline phosphatase (ALPL), and the osteogenic late stage markers osteopontin (SPP1) and osteocalcin (BGLAP) were analysed by RT-qPCR after 7 and 14 days of mechanical stimulation. The undifferentiated and mechanically unstimulated group (EX(−)) served as the reference value (ratio = 1). RUNX2 mRNA levels showed a clear, but not statistically significant, increase after 14 days of mechanical stimulation. Following 7 and 14 days of cyclic strain, the expression of Col1A1 increased significantly in the mechanically stimulated OG cells at both time points. The expression of the late stage marker SPP1 increased significantly during osteoinduction in OG(+) cells. However, no significant differences between the four groups were indicated in the mRNA expression of BGLAP. The most significant increase was observed by the BMP2 expression. SPARC expression was increased in the osteogenic differentiated mechanically unstimulated group OG(−) at day 7 and in the OG(+) cells at day 14 when compared to the EX(−) controls. After both time points, a significant increase in ALDL expression was observed in the OG(−) and OG(+) cells. All values are listed in Table 2.

### 3.5. Mechanical Stimulation Enhanced the Expression of Osteogenesis-Specific Markers

**Figure 3:**
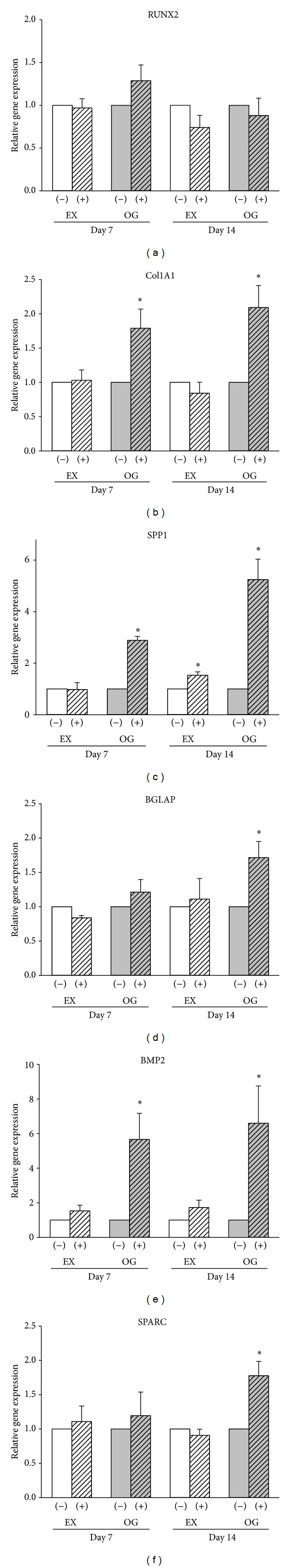
Regulation of osteogenesis-specific markers under the influence of mechanical stimulation. mRNA levels of (a) RUNX2, (b) Col1A1, (c) SPP1, (d) BGLAP, (e) BMP2, and (f) SPARC were normalized to their respective mechanically unstimulated control groups. EX(−) represents the undifferentiated mechanically unstimulated group, EX(+) the undifferentiated mechanically stimulated group, OG(−) the osteogenic differentiated mechanically unstimulated group, and OG(+) the osteogenic differentiated mechanically stimulated group. The values are mean ± SD of independent experiments performed in triplicate (*n* = 7); *P < 0.05.

To investigate the influence of the mechanical stimulation on both undifferentiated and osteogenic differentiated intraoral MSPCs, RT-qPCR values were subjected to a further evaluation. Mechanically stimulated cells were compared to the respective unstimulated control cells. Whereas the undifferentiated (EX) intraoral MSPCs showed no change in the osteogenesis-specific marker expression, mechanical stimulation increased the expression of Col1A1, SPP1, BGLAP, BMP2, and SPARC significantly in the osteogenic differentiated (OG) cells. Specifically, whereas no significant differences between cyclic strain and static condition could be observed by RUNX2 (Figure 3(a)), Col1A1 mRNA levels increased to 1.79 ± 0.73 (*P* = 0.029) after 7 days and to 2.09 ± 0.71 (*P* = 0.026) after 14 days of mechanical stimulation (Figure 3(b)). SPP1 increased significantly in the mechanically stimulated OG cells to 2.88 ± 0.34 (*P* = 0.0002) at day 7 and 5.25 ± 1.37 (*P* = 0.033) at day 14 (Figure 3(c)). BGLAP expression considerably to 1.72 ± 0.52 (*P* = 0.037) in OG(+) cells exposed to 14 days of mechanical strain (Figure 3(d)). The most significant increase was observed in the BMP2 expression after 7 days (5.66 ± 3.98; *P* = 0.021) and 14 days (6.60 ± 4.82; *P* = 0.048) of mechanical stimulation (Figure 3(e)). SPARC expression increased significantly to 1.77 ± 0.46 (*P* = 0.020) in OG(+) cells exposed to 14 days of mechanical strain (Figure 3(f))

**Figure 4:**
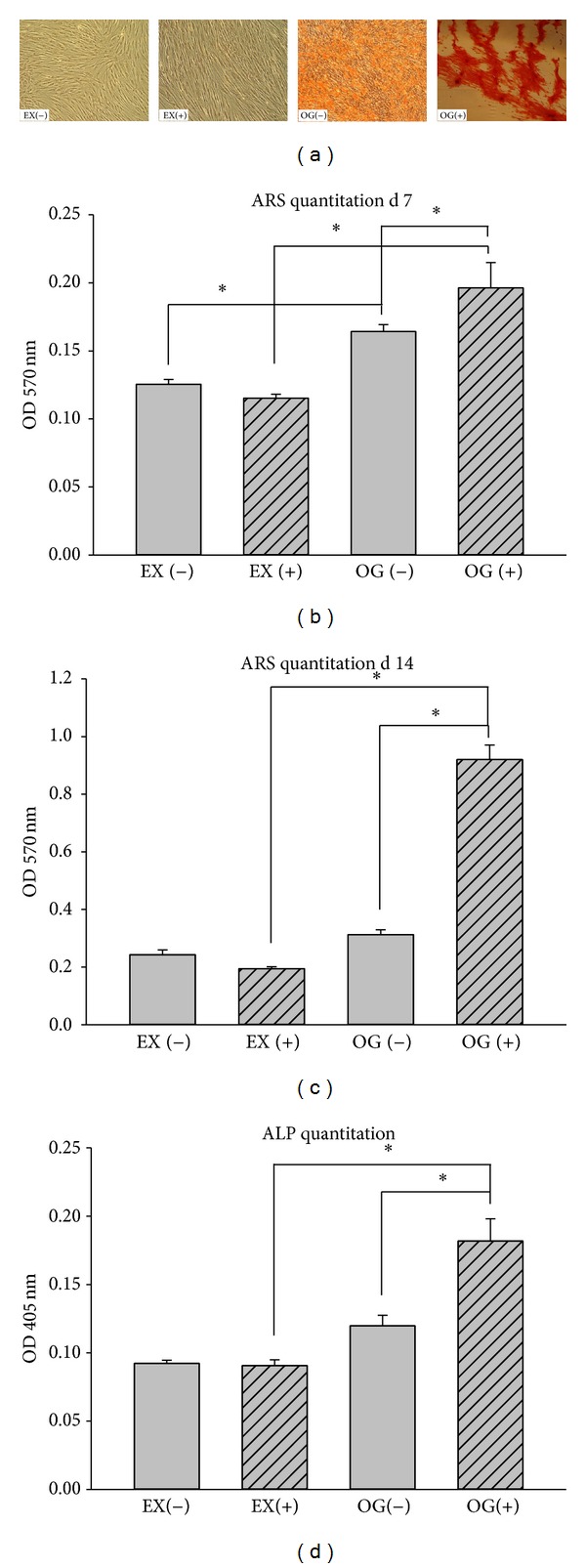
Influence of mechanical stimulation on alkaline phosphatase (ALP) expression and demonstration of mineralized matrix using ARS staining of human intraoral MSPCs. (a) Representative pictures of unstained (EX(−) and EX(+)) and osteogenic differentiated (OG(−) and OG(+)) MSPCs. Quantitation of calcium deposited into the cultures revealed a significant increase in the 570 nm OD in osteogenic differentiated and mechanically stimulated MSPCs after (b) 7 days and (c) 14 days, when compared to undifferentiated controls. (d) Quantitation of ALP enzymes revealed a significant increase compared to corresponding controls. EX(−) represents the undifferentiated mechanically unstimulated group, EX(+) the undifferentiated mechanically stimulated group, OG(−) the osteogenic differentiated mechanically unstimulated group, and OG(+) the osteogenic differentiated mechanically stimulated group. The values are mean ± SD of independent experiments performed in triplicate (*n* = 9); *P < 0.05.

In addition, ARS staining of calcium deposits and absorbance measurements of ARS-stained intraoral MSPCs eluted with cetylpyridinium chloride at an optical density (OD) of 570 nm were performed at days 7 and 14. Representative pictures of unstained (EX(−) and EX(+)) and osteogenic differentiated (OG(−) and OG(+)) MSPCs are shown in Figure 4(a). Values measured at an OD of 570 nm increased significantly over time in osteogenic lineage cells when compared to undifferentiated controls. Corresponding to the RT-qPCR data, mechanical stimulation revealed a significant increase of quantifiable calcium deposits in the OG(−) cells compared to the OG(+) cells (Figures 4(b) and 4(c)). ALP activity was measured over the absorbance of p-nitrophenol phosphate, a chromogenic product with absorbance at 405 nm, in supernatant after 14 days. Again, cyclic strain enhanced the activity of ALP in the OG(+) cells compared to the OG(−) cells (*P* = 0.0052) (Figure 4(d)). All values are listed in Table 3.

## 4. Discussion

To date, most experimental and clinical tissue engineering trials in craniofacial surgery have used BM-derived stromal cells from the iliac crest [24, 25]. In a previous work we demonstrated that cells isolated from mandibular and maxillary bone, periosteum from the oblique line, and dental pulp exhibit the characteristics of cells described as MSPCs [7]. For the first time, the role of mechanical stimulation in osteogenic differentiated intraoral MSPCs is the subject of investigation. Consequently, explant cultures of human MSPCs have been established from human intraoral tissue samples of posterior maxilla and mandibular retromolar bone and characterized as MSPCs according to the criteria of the International Society for Cellular Therapy [26] using flow cytometry analysis. The low number of CD45-positive cells indicates that the bone-derived cultures contain immature mesenchymal cells. The small number of CD45-positive cells was reported in the surface profile of adult MSPCs [27]. Yu et al. demonstrated these small CD45-positive subpopulations in adipose tissue-derived MSPCs as well [28].

*In vitro*, bone cells demonstrate a high responsiveness to mechanical stimuli. Much debate exists regarding the critical components of the load profile and whether different components, such as fluid shear, tension, or compression, influence cells differently. The most widely used mechanical stimuli* in vitro* are cyclic stretch and fluid shear flow [29]. We seeded the intraoral MSPCs onto collagen type-I-coated BioFlex plates and stimulated them mechanically using a continuous uniaxial sinusoidal waveform with 10% elongation and a frequency of 0.5 Hz.

LDH release and the expression of osteogenesis-specific markers were analysed after 7 and 14 days. The effect of mechanical loading on the proliferation of osteoblastic cells is controversial. Some studies have shown that an appropriate amount of mechanical force can induce the growth of BM-MSPCs [30], while others have found the opposite [31]. In our experiments, the relative levels of LDH in the 0% and 10% strained groups showed no significant changes. These findings suggest that 10% cyclic strain did not change the survival rate of intraoral MSPCs or induce serious cellular damage.

Similar to the results on rat BM-MSPCs reported by Zhao et al. [32], our RT-qPCR data showed significantly increased mRNA expression levels of type-I collagen (Col1A1), osteonectin (SPARC), bone morphogenetic protein 2 (BMP2), alkaline phosphatase (ALPL), osteopontin (SPP1), and osteocalcin (BGLAP) in the mechanically stimulated groups. The runt-related transcription factor 2 (RUNX2) is considered to be the central control gene within the osteoblast phenotype. Xiao et al. have shown that levels of RUNX2 during* in vitro* differentiation of primary human osteoblasts showed no major changes, whereas levels of downstream genes such as bone sialoprotein and alkaline phosphatase were dramatically increased [32, 33]. Furthermore, real-time PCR and western blot analyses indicated that there was no significant increase in the amount of RUNX2 protein or mRNA during human osteoblast differentiation [34].

However, although the expression level of RUNX2 showed no significant differences in human intraoral MSPCs, our RT-qPCR data showed significantly increased mRNA expression levels of the RUNX2 downstream genes Col1A1, ALPL, SPP1, and BGLAP in the mechanically stimulated groups. The most prominent difference we observed between unstimulated and mechanically stimulated, osteogenic differentiated MSCPs was in the expression of BMP2. This result is similar to the findings of Sumanasinghe et al., who demonstrated that BMP2 mRNA is significantly increased in strained groups [16]. The late stage markers SPP1 and BGLAP play an important role in the differentiation of osteoblast progenitor cells, with significant upregulation observed in both matrix synthesis and mineralisation. Our results are in accordance with other studies, which demonstrated an upregulation of these genes with steady increases as osteoblastic differentiation progresses [34, 35]. Both ALP activity and the production of mineralized matrix were significantly upregulated by mechanical stimulation in our human intraoral MSPCs. The production of mineralised matrix is considered a marker for terminally differentiated MSPCs into osteoblast-like cells [36]. Therefore, mineral formation is an appropriate indicator underpinning the fact that mechanical stimulation accelerates the osteogenic differentiation of MSPCs. The protein levels have also been shown to be upregulated in response to the application of mechanical force [34].

## 5. Conclusion

Our results revealed that 10% cyclic strain induced a significant increase in the mRNA expression of the osteogenesis-specific markers in osteogenic differentiated intraoral MSPCs. The increasing evidence for mechanical stimulation as a regulator of osteogenic differentiation in MSPCs holds important consequences for the development of craniofacial surgery and orthopaedic tissue engineering solutions. Further, molecular mechanisms underlying cellular responses to mechanical stimulation are not well understood. Thus, further investigation is necessary to better understand the molecular mechanism underlying the effects of mechanical stimulation on the osteogenic differentiation of human intraoral MSPCs.

## Figures and Tables

**Table 1:** Expression of intraoral MSPC surface proteins analysed by flow cytometry.

Cluster of differentiation (CD)	Positivity (%)
CD73 PE	99.8 ± 0.1
CD90 APC	99.9 ± 0.1
CD105 PerCP-Cy5.5	69.1 ± 9.8
CD14 FITC	0.2 ± 0.2
CD19 APC	0.6 ± 0.1
CD34 FITC	0.4 ± 0.3
CD45 APC-Cy7	23.9 ± 7.8
HLA-DR PerCP-Cy5.5	0.5 ± 0.3

Expression of intraoral MSPC surface proteins analysed by flow cytometry. Mean values of the percentage of positive cells ± SD to the total number of analysed cells are shown (*n* = 10). PE: phycoerythrin; APC: allophycocyanin; PerCP: peridium-chlorophyll protein complex; FITC: fluorescein isothiocyanate.

**Table 2:** Expression of the osteogenesis-specific markers affected by mechanical stimulation for 7 and 14 days.

	Day 7				Day 14			
	EX(−)	EX(+)	OG(−)	OG(+)	EX(−)	EX(+)	OG(−)	OG(+)
RUNX2	1	1.01 ± 0.3	0.98 ± 0.3	1.40 ± 0.5	1	0.75 ± 0.3	2.48 ± 0.6	3.13 ± 1.1
Col1A1	1	1.16 ± 0.3	1.70 ± 0.8	2.92 ± 1.1 *P = 0.0039 *	1	0.68 ± 0.4	0.94 ± 0.4	2.02 ± 0.9 *P = 0.049 *
SPP1	1	0.95 ± 0.3	1.11 ± 0.4	1.91 ± 0.5 *P = 0.040 *	1	1.44 ± 0.7	1.48 ± 0.2	2.92 ± 0.8 *P = 0.038 *
BGLAP	1	0.85 ± 0.1	0.78 ± 0.4	0.84 ± 0.5	1	0.67 ± 0.2	0.91 ± 0.5	1.08 ± 0.2
BMP2	1	1.53 ± 0.8	2.17 ± 1.3	5.54 ± 3.2 *P = 0.036 *	1	2.06 ± 1.0	3.84 ± 1.8	6.48 ± 3.5 *P = 0.013 *
SPARC	1	0.93 ± 0.2	1.47 ± 0.4 *P = 0.035 *	1.60 ± 0.9	1	0.88 ± 0.3	0.95 ± 0.4	2.13 ± 0.8 *P = 0.044 *
ALPL	1	0.86 ± 0.4	5.35 ± 1.9 *P = 0.042 *	4.05 ± 1.8 *P = 0.046 *	1	0.78 ± 0.5	3.55 ± 1.6 *P = 0.023 *	3.62 ± 1.8 *P = 0.042 *

mRNA levels of RUNX2, Col1A1, SPP1, and BGLAP. BMP2 and ALPL were normalized to the undifferentiated mechanically unstimulated control group EX(−) of each day (ratio = 1). EX(+) represents the undifferentiated mechanically stimulated group, OG(−) the osteogenic differentiated mechanically unstimulated group, and OG(+) the osteogenic differentiated mechanically stimulated group. The values are mean ± SD of independent experiments performed in triplicate (*n* = 7); *P < 0.05.

**Table 3:** Quantitation of ARS staining of calcium deposits and ALP enzyme.

	EX(−)	EX(+)	OG(−)	OG(+)
ARS day 7	0.12 ± 0.01	0.11 ± 0.01	0.16 ± 0.02	0.20 ± 0.05
EX versus OG			*P = 2.15E − 05 *	*P = 0.002 *
(−) versus (+)				*P = 0.037 *

ARS day 14	0.24 ± 0.06	0.20 ± 0.02	0.31 ± 0.06	0.92 ± 0.19
EX versus OG			*P = 0.007 *	*P = 6.56E − 10 *
(−) versus (+)				*P = 2.02E − 9 *

ALP day 14	0.09 ± 0.01	0.09 ± 0.01	0.12 ± 0.02	0.18 ± 0.04
EX versus OG				*P = 0.0004 *
(−) versus (+)				*P = 0.005 *

The ARS staining of calcium deposits and measurements of an OD of 570 nm after cetylpyridinium chloride elution were performed at days 7 and 14. Quantitation of calcium deposits revealed a highly significant increase of an OD of 570 nm values in osteogenic differentiated cells (OG) compared to the corresponding undifferentiated controls (EX). Mechanical stimulation revealed a significant increase in calcium deposits in the OG(−) cells compared to the OG(+) cells. Quantitation of ALP enzyme at day 14 revealed a significant increase in osteogenic differentiated cells (OG) compared to the corresponding undifferentiated controls (EX). The values are mean ± SD of independent experiments performed in triplicate (*n* = 12); *P < 0.05.
